# Supplementary material for: Quantification of the overall contribution of gene-environment interaction for obesity-related traits
Source: Nat Commun. 2020 Mar 13;11:1385. doi: 10.1038/s41467-020-15107-0 (PMC7070002; doi:10.1038/s41467-020-15107-0)
Supplement: Supplementary file 1 — Supplementary Information New [file 41467_2020_15107_MOESM1_ESM.pdf]

Supplementary Information: Quantification of the overall contribution of gene-environment interaction for obesity-related traits

Jonathan Sulc[1,2,*], Ninon Mounier[1,2,*], Felix, Günther[3], Thomas Winkler[3],Andrew R. Wood[4], Timothy M. Frayling[4], Iris M. Heid[3], Matthew R. Robinson[5], Zoltán Kutalik[1,2,4,†]

Supplementary Notes

Supplementary Note 1: Likelihood function for binary outcome

For analytical simplicity, assuming an interaction probit model implies that

$$Pr(y_i = 1 | g_i, e_i) \quad = \quad \prod_{i=1}^{n} \Phi\left(\frac{\alpha g_i + \beta e_i + \gamma(e_i * g_i) - z_0}{\sigma}\right) \quad \text{with} \quad z_0 = \Phi^{-1}(1-\pi)$$

where $\pi = \sum_i y_i/n$ is the prevalence of the disease. If the environment is not available the probability modifies as follows

$$\begin{aligned} Pr(y_i = 1|g_i) &= \int_{-\infty}^{\infty} Pr(y_i = 1|g_i, e_i) Pr(e_i) de_i \\ &= \int_{-\infty}^{\infty} \Phi\left(\frac{\alpha g_i + \beta e_i + \gamma(e_i * g_i) - z_0}{\sigma}\right) \phi(e_i) de_i \\ &= \Phi\left(\frac{\alpha g_i - z_0}{\sqrt{(\beta + \gamma g_i)^2 + \sigma^2}}\right) \end{aligned}$$

using the integral identity $\int \Phi(a + bx) \cdot \phi(c + dx)dx = \Phi((ad - bc)/\sqrt{b^2 + d^2})/d$.

The log likelihood function therefore can be written as

$$\begin{aligned} l(\alpha, \beta, \gamma, \sigma) &= Pr(\boldsymbol{y}, \boldsymbol{g}|\alpha, \beta, \gamma, \sigma) \\ &= \sum_{y_i=1} \log\left(\Phi\left(\frac{\alpha g_i - z_0}{\sqrt{(\beta + \gamma g_i)^2 + \sigma^2}}\right)\right) + \sum_{y_i=0} \log\left(\Phi\left(-\frac{\alpha g_i - z_0}{\sqrt{(\beta + \gamma g_i)^2 + \sigma^2}}\right)\right) \end{aligned}$$

[1]University Center for Primary Care and Public Health, University of Lausanne, 1010, Switzerland

[2]Swiss Institute of Bioinformatics, Lausanne, 1015, Switzerland

[3]Department of Genetic Epidemiology, University of Regensburg, Regensburg, Germany

[4]Genetics of Complex Traits, University of Exeter Medical School, University of Exeter, UK

[5]Department of Computational Biology, University of Lausanne, 1015, Lausanne, Switzerland

[*]These authors contributed equally to this work.

[†]Correspondance should be addressed to zoltan.kutalik@unil.ch

Supplementary Note 2: Violations of the marginal-interaction effect concordance

Let us assume that all variables Y, G_j, E_k are standardised and the true underlying model is

$$\begin{aligned} Y &= \sum_j a_j \cdot G_j + \sum_k b_k \cdot E_k + \sum_j \sum_k c_{j,k} \cdot (G_j \cdot E_k) + \epsilon \\ &= \sum_j a_j \cdot G_j + \sum_k b_k \cdot E_k + \sum_j \sum_k (a_j \cdot r_k + \eta_{j,k}) \cdot (G_j \cdot E_k) \\ &= \sum_j a_j \cdot G_j + \sum_k b_k \cdot E_k + \sum_j a_j G_j \sum_k (r_k E_k) + \sum_j G_j \sum_k \eta_{j,k} \cdot E_k \end{aligned}$$

with r_k is the coefficient from regressing $\boldsymbol{a}$ onto $\boldsymbol{c_k} := c_{\cdot,k}$, i.e. $r_k = corr(\boldsymbol{a}, \boldsymbol{c_k}) \cdot \sqrt{Var(\boldsymbol{c_k})/Var(\boldsymbol{a})}$. We however fit the model

$$Y = \alpha G + \beta E + \gamma(G \cdot E) + \epsilon \qquad \text{where} \quad G = \frac{\sum_j a_j \cdot G_j}{\sqrt{\sum_j a_j^2}} \quad \text{and} \quad E = \frac{\sum_k r_k E_k}{\sqrt{\sum_k r_k^2}}$$

Substituting these regressors G, E into the true underlying model stated above we get

$$\begin{aligned} Y &= \alpha \cdot G + \sum_k b_k \cdot E_k + \left(\sqrt{\sum_j a_j^2} \cdot \sqrt{\sum_k r_k^2} \right) G \cdot E + \sum_j G_j \sum_k \eta_{j,k} \cdot E_k \\ &= \alpha \cdot G + corr(\boldsymbol{r}, \boldsymbol{b}) \sqrt{\sum_k b_k^2} \cdot E + \left(\sqrt{\sum_j a_j^2} \cdot \sqrt{\sum_k r_k^2} \right) G \cdot E + \tau \\ &= \alpha \cdot G + corr(\boldsymbol{r}, \boldsymbol{b}) \sqrt{\sum_k b_k^2} \cdot E + \left(\alpha \cdot \sqrt{\sum_k r_k^2} \right) G \cdot E + \tau \\ &= \alpha \cdot G + corr(\boldsymbol{r}, \boldsymbol{b}) \sqrt{\sum_k b_k^2} \cdot E + \left(\alpha \cdot \sqrt{\sum_k corr(\boldsymbol{a}, \boldsymbol{c_k})^2 \cdot Var(\boldsymbol{c_k})/Var(\boldsymbol{a})} \right) G \cdot E + \tau \\ &= \alpha \cdot G + \underbrace{corr(\boldsymbol{r}, \boldsymbol{b}) \sqrt{\sum_k b_k^2}}_{\beta} \cdot E + \underbrace{\sqrt{\sum_k corr(\boldsymbol{a}, \boldsymbol{c_k})^2 \cdot Var(\boldsymbol{c_k})}}_{\gamma} \cdot G \cdot E + \tau \end{aligned}$$

Which means that the total variance explained by the interaction captured by the simplified model is

$$\frac{\sum_k corr(\boldsymbol{a}, \boldsymbol{c_k})^2 \cdot Var(\boldsymbol{c_k})}{\sum_{j,k} c_{j,k}^2}$$

which reached the maximum of 1 when the interaction effect of all environmental factors E_k is perfectly correlated with the marginal effects of SNPs.

Supplementary Note 3: Likelihood function in case of low/no interaction effect

Let us assume that the underlying true model has no interaction ($\gamma = 0$), i.e. $Y = \alpha_1 \cdot G + \alpha_2 \cdot G^2 + \epsilon$. The likelihood function for data generated under this model is of the form

$$\begin{aligned}
\log \mathcal{L}(\theta|\boldsymbol{y}, \boldsymbol{g}) &= -\frac{1}{2} \times \sum_i \log\left((\beta + \gamma \cdot g_i)^2 + \sigma^2\right) + \left(\frac{(y_i - \alpha_1 \cdot g_i - \alpha_2 \cdot (g_i^2 - 1))^2}{(\beta + \gamma \cdot g_i)^2 + \sigma^2}\right) \\
&= -\frac{1}{2} \times \sum_i \log\left((\beta + \gamma \cdot g_i)^2 + \sigma^2\right) + \left(\frac{\epsilon_i^2}{(\beta + \gamma \cdot g_i)^2 + \sigma^2}\right)
\end{aligned}$$

with $\epsilon_i \sim \mathcal{N}(0, 1 - \alpha_1^2 - 2\alpha_2^2)$, $g_i \sim \mathcal{N}(0,1)$, $\sigma^2 = 1 - \alpha_1^2 - 2\alpha_2^2 - \beta^2 - \gamma^2$. The derivative with respect to σ then simplifies to

$$\frac{\partial}{\partial \sigma} \log \mathcal{L}(\theta|\boldsymbol{y}, \boldsymbol{g}) = -\frac{1}{2} \cdot \sum_{i=1}^{n} \left(\frac{2\sigma}{(\beta + \gamma \cdot g_i)^2 + \sigma^2} - \frac{2\epsilon_i^2 \cdot \sigma}{((\beta + \gamma \cdot g_i)^2 + \sigma^2)^2}\right)$$

Hence as γ goes to zero we have

$$\lim_{\gamma \to 0} \frac{\partial}{\partial \sigma} \log \mathcal{L}(\theta|\boldsymbol{y}, \boldsymbol{g}) = \sigma \cdot n \cdot \frac{1}{(\beta^2 + \sigma^2)^2} \cdot \left(\frac{\sum_{i=1}^n \epsilon_i^2}{n} - (\beta^2 + \sigma^2)\right)$$

where $E(\epsilon_i^2) = \beta^2 + \sigma^2$. Therefore, the expectation of the last term is zero, and its limit (as $n \to \infty$) is zero, but it does not depend on σ, by construction, for finite n. For this reason, the partial derivative is zero only if $\sigma^2 = 0$. This explains why the optimum of the likelihood function ends up on the boundary (characterised by $\sigma^2 = 0$) when γ is approaching zero. To confirm that it is indeed the maximum (and not a minimum), the second derivative is

$$\lim_{\gamma \to 0} \frac{\partial^2}{\partial \sigma^2} \log \mathcal{L}(\theta|\boldsymbol{y}, \boldsymbol{g}) = n \cdot \frac{1}{(\beta^2 + \sigma^2)^3} \cdot \left(\frac{\sum_{i=1}^n \epsilon_i^2}{n} \cdot \left(\beta^2 - 3\sigma^2\right) + (\sigma^4 - \beta^4)\right)$$

Thus

$$E\left[\lim_{\gamma \to 0} \frac{\partial^2}{\partial \sigma^2} \log \mathcal{L}(\theta|\boldsymbol{y}, \boldsymbol{g})\right] = n \cdot \frac{1}{(\beta^2 + \sigma^2)^3} \cdot \left(-2\sigma^2 \left(\beta^2 + \sigma^2\right)\right) = \frac{-2n\sigma^2}{(\beta^2 + \sigma^2)^2} < 0 \ .$$

Supplementary Note 4: Bias in interaction parameter estimation upon transformation

Let us assume that trait Z can be written as $\alpha G + \beta E + \gamma(G \times E) + \epsilon$. If we model, instead of Z, a transformed version of it $f(Z)$, the MLE estimation will be biased. As we have shown in the Methods section of the main paper, to the new interaction effect for $f(Z)$, we need to estimate the coefficient of g^2 for $Var(f(Z)|G = g)$. For this we can use the second order Taylor series expansion of $f(\cdot)$ around zero.

$$
\begin{aligned}
Var(f(Z)|G=g) &= E((f(Z))^2|G=g) - E^2(f(Z)|G=g) \\
&= E\left(\left[f(0) + f'(0)(\alpha g + \beta E + \gamma(g \times E) + \epsilon) + (1/2)\cdot f''(0)(\alpha g + \beta E + \gamma(g \times E) + \epsilon)^2\right]^2\right) \\
&- E^2(f(0) + f'(0)(\alpha g + \beta E + \gamma(g \times E) + \epsilon) + (1/2)\cdot f''(0)(\alpha g + \beta E + \gamma(g \times E) + \epsilon)^2)
\end{aligned}
$$

The coefficient of g^2 of the first term (i.e. $E((f(Z))^2|G = g)$) is

$$
\begin{aligned}
& 3f'(0)\beta E^3 f''(0)\gamma^2 + 3f''(0)^2\alpha G\gamma^2 E^2\epsilon + f(0)f''(0)\gamma^2 E^2 + f'(0)^2\gamma^2 E^2 + (3/2)f''(0)^2\alpha^2 G^2\gamma^2 E^2 \\
+ & 3f''(0)^2\alpha G\beta E^3\gamma^2 + 3f''(0)^2\beta E^3\gamma^2\epsilon + (3/2)f''(0)^2\gamma^2 E^2\epsilon^2 \\
+ & (3/2)f''(0)^2\beta^2 E^4\gamma^2 + 3f'(0)\gamma^2 E^2 f''(0)\epsilon + 3f'(0)\alpha G f''(0)\gamma^2 E^2
\end{aligned}
$$

Its expectation is

$$
\begin{aligned}
& 3f'(0)f''(0)\beta\gamma^2 K_3 + f(0)f''(0)\gamma^2 + f'(0)^2\gamma^2 + (3/2)f''(0)^2\alpha^2\gamma^2 \\
+ & (3/2)f''(0)^2\gamma^2\sigma^2 + (3/2)f''(0)^2\beta^2\gamma^2 K_4 \\
= & 3f'(0)f''(0)\beta\gamma^2 K_3 + f(0)f''(0)\gamma^2\sigma^2 + f'(0)^2\gamma^2 + (3/2)f''(0)^2\gamma^2(\alpha^2 + \sigma^2 + \beta^2 K_4)
\end{aligned}
$$

where $K_3 = E[E^3]$ and $K_4 = E[E^4]$ are the skewness and kurtosis of E. The squared expectation of $(f(Z)|G = g)$ is

$$
E^2(f(Z)|G=g) = \left((1/2)f''(0)(\beta + \gamma\cdot g)^2 + (1/2)f''(0)\alpha^2 + f(0) + (1/2)f''(0)\sigma^2\right)^2
$$

The coefficient of g^2 of the square of this expression is

$$
\left((1/2)f''(0)\beta^2 + (1/2)f''(0)\sigma^2 + (1/2)f''(0)\alpha^2 + f(0)\right) f''(0)\gamma^2 + f''(0)^2\beta^2\gamma^2
$$

Therefore, the coefficient of the g^2 term of $Var(f(Z)|G = g)$ is approximately

$$
\begin{aligned}
\gamma^\star &\approx 3f'(0)f''(0)\beta\gamma^2 K_3 + f(0)f''(0)\gamma^2 + f'(0)^2\gamma^2 + (3/2)f''(0)^2\gamma^2(\alpha^2 + \sigma^2 + \beta^2 K_4) \\
&- \left(((1/2)f''(0)\beta^2 + (1/2)f''(0)\sigma^2 + (1/2)f''(0)\alpha^2 + f(0))f''(0)\gamma^2 + f''(0)^2\beta^2\gamma^2\right) \\
&= \gamma^2\cdot\left(3f'(0)f''(0)\beta K_3 + f'(0)^2 + f''(0)^2(\alpha^2 + \sigma^2 + (3/2)\beta^2(K_4 - 1))\right)
\end{aligned}
$$

This gives an approximate analytical formula for the bias introduced in the interaction effect estimation upon trait transformation. Note that the formula indicates that with larger skewness and kurtosis, the bias increases. It can be observed that if $f''(0) = 0$, i.e. only a linear transformation is applied, the expression simplifies to $f'(0)^2\cdot\gamma$.

Supplementary Figures

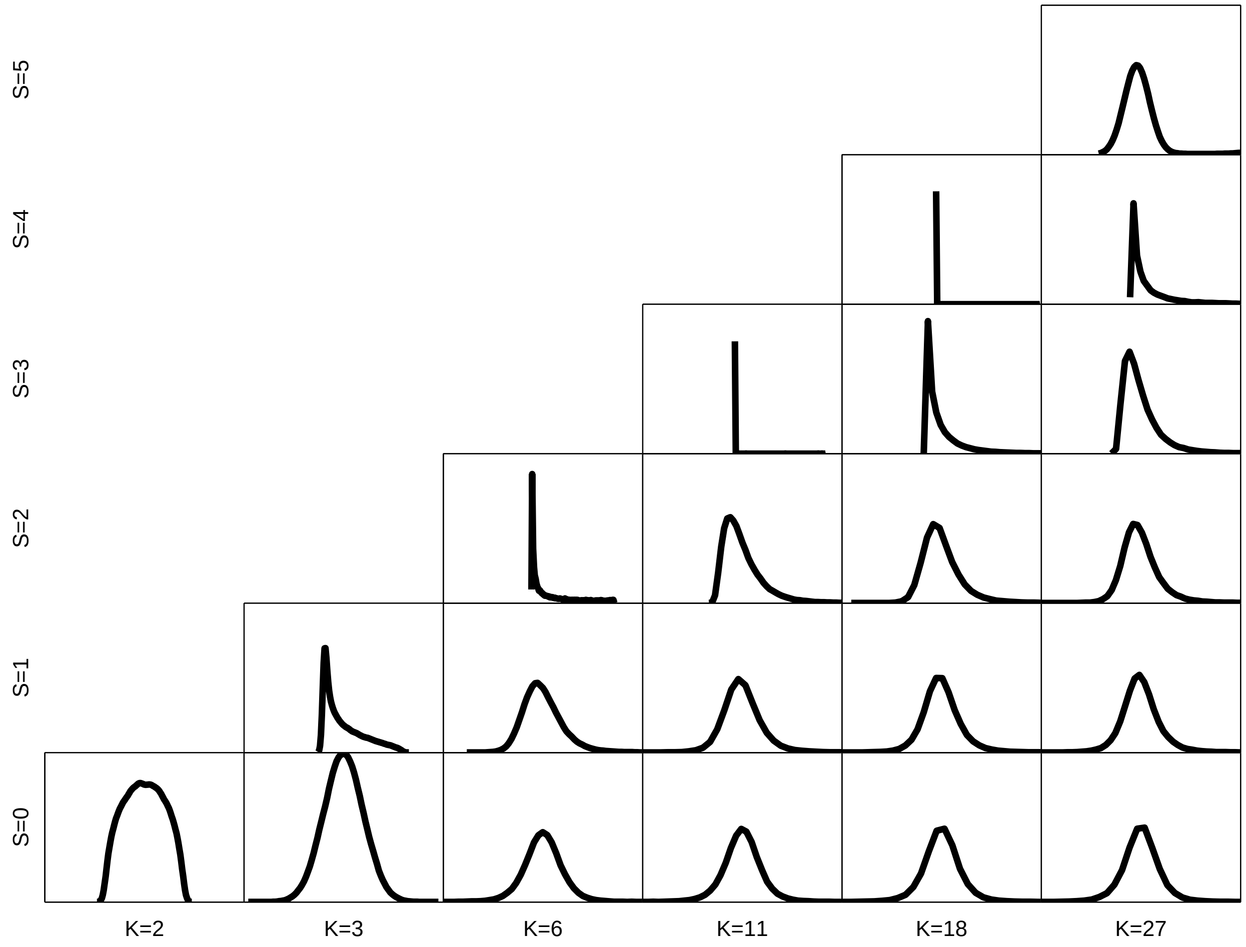

Supplementary Figure 1: Density functions of various combinations of skewness and kurtosis. K=kurtosis, S=skewness. Note that K=3, S=0 corresponds to the Gaussian distribution.

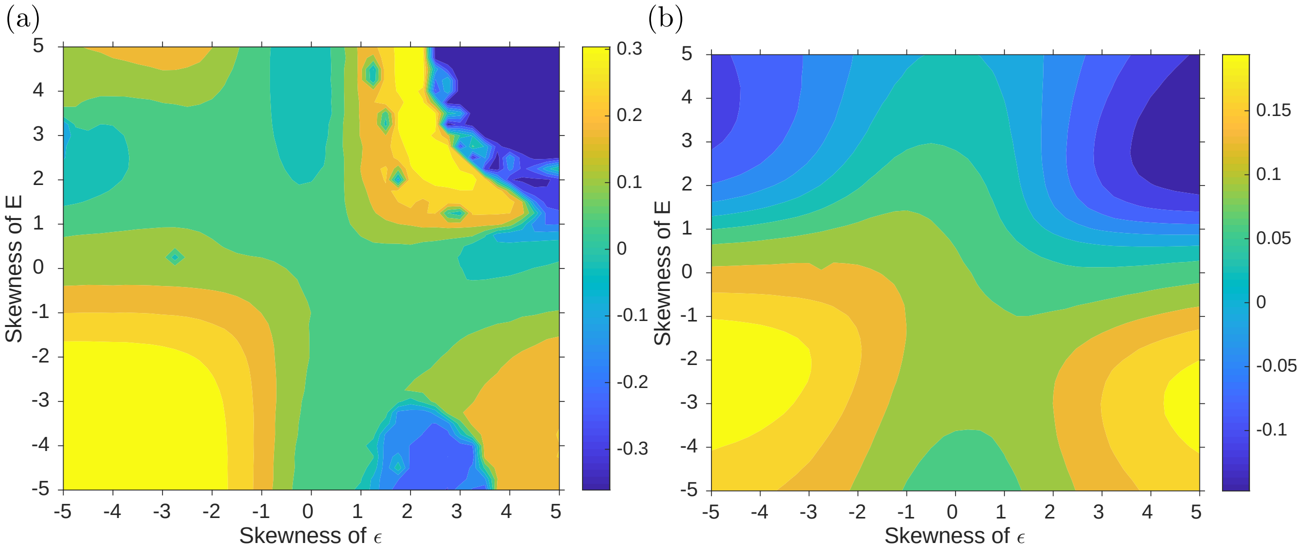

Supplementary Figure 2: Interaction effect estimates for INQT traits. The original trait was generated by the model $Y = \sqrt{0.05}G + \sqrt{0.3}E + \sqrt{0.01}(G \times E) + \epsilon$ for all possible skewness combinations for E and ϵ in the range of $(-5, 5)$ and kurtosis set to $skewness^2 + 3$, while n was set to 100,000. Panel (a): Such Y was then inverse normal quantile transformed and subjected to our MLE method. While for the untransformed trait our method correctly returned an interaction estimate very close to the true value ($\gamma = 0.1$), the INQT(Y) can produce arbitrary bias depending on the skewness of E and ϵ. Panel (b): Same analysis results when assuming E is known and applying $Y \sim G + E + (G \times E)$ model via linear regression.

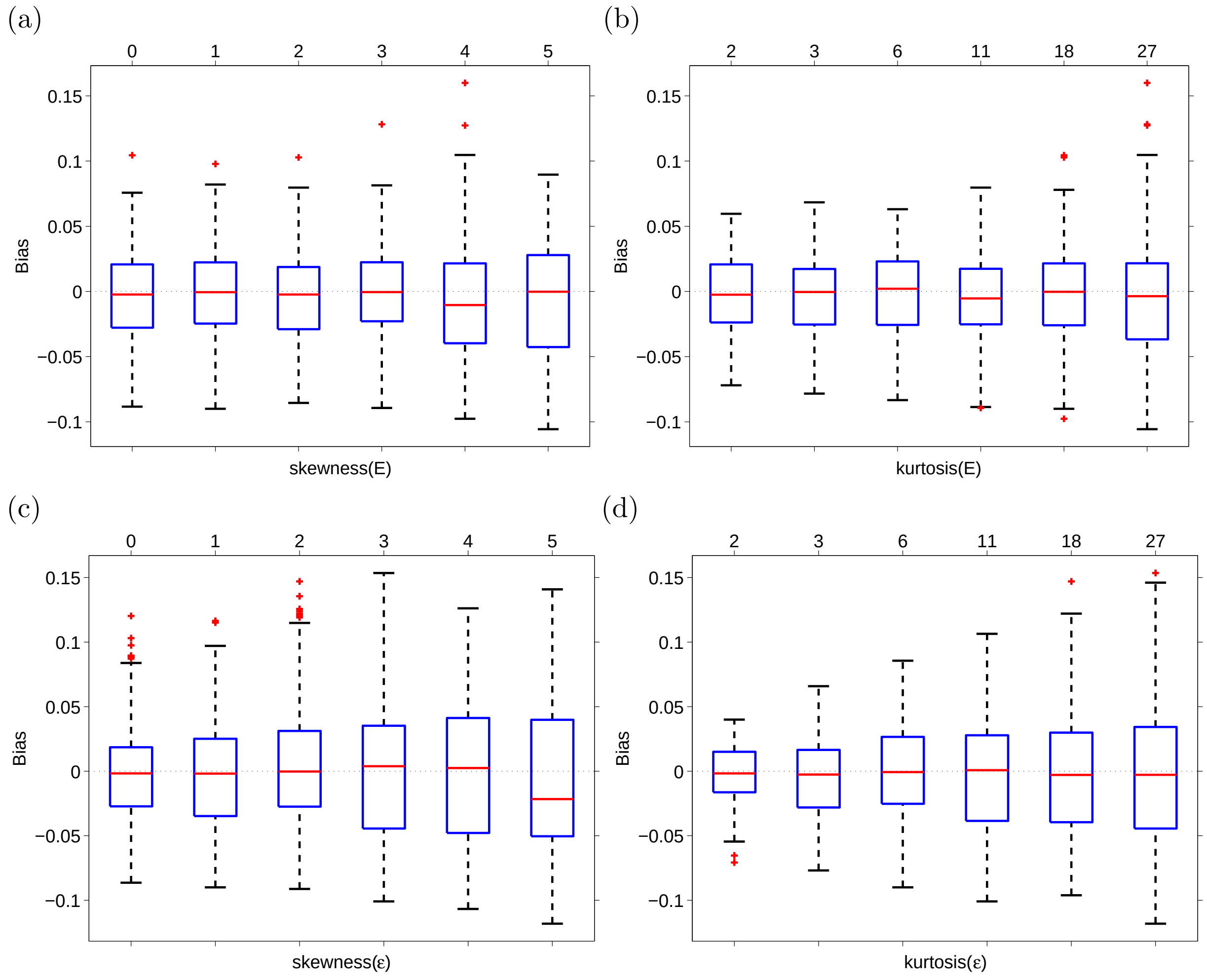

Supplementary Figure 3: Interaction effect estimation bias as a function of skewness and kurtosis of the environmental variable (E - panels (a-b)) or the noise (ϵ - panels (c-d)). Parameters were fixed as $n = 10,000$, $\alpha_1^2 = 0.1$, $\alpha_2 = 0$, $\beta^2 = 0.3$, $\gamma^2 = 0.025$. Boxes mark the first (q_1) second (q_2) and third quartiles (q_3) and the lower/upper whiskers are at $q_1 - 1.5 \cdot (q_3 - q_1)$, $q_3 + 1.5 \cdot (q_3 - q_1)$, respectively.

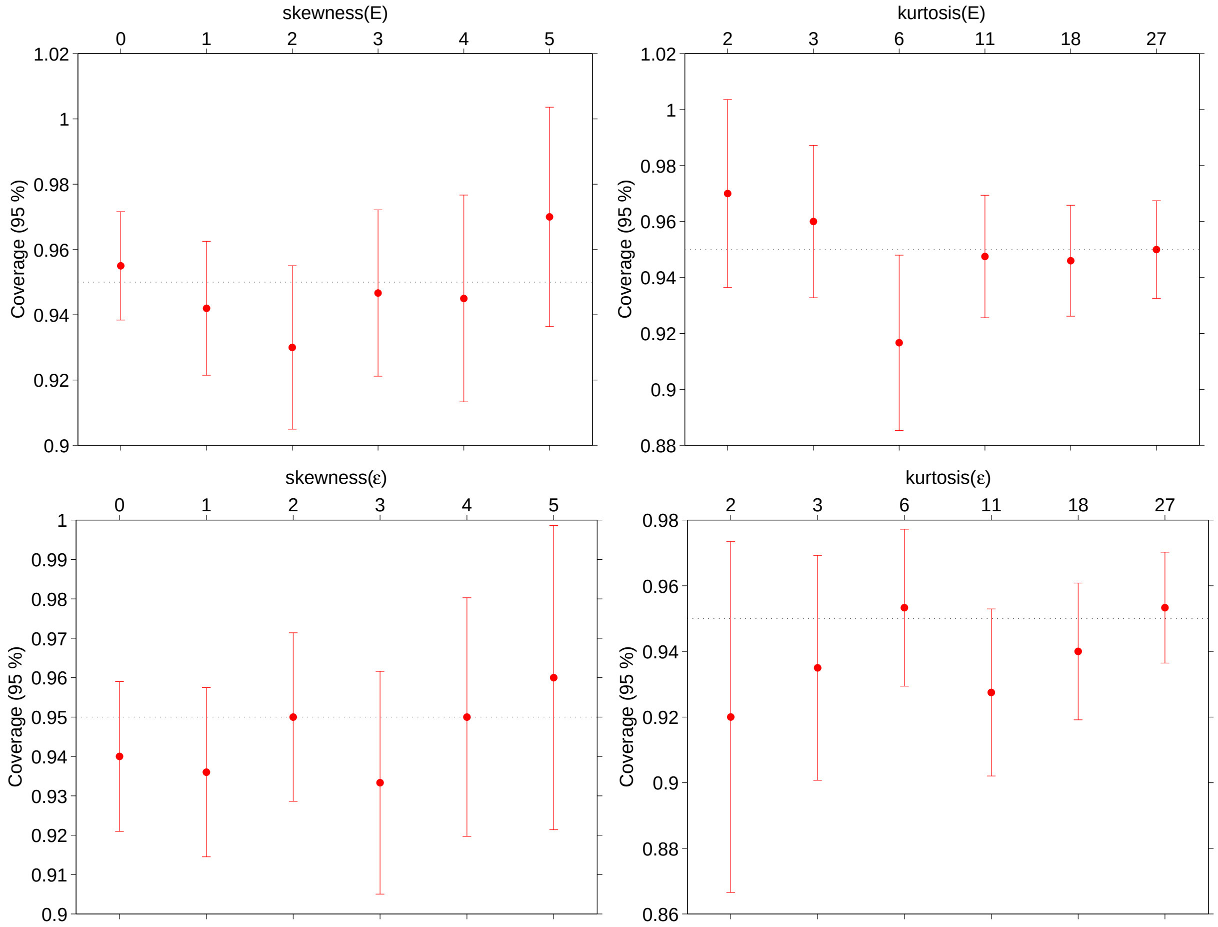

Supplementary Figure 4: Coverage of the 95% confidence interval (i.e. the proportion of simulations where the true parameter value falls into the 95% confidence interval) as a function of skewness and kurtosis of the environmental variable (E - top panels) or the noise (ε - bottom panels). Parameters were fixed as above: $n = 10,000, \alpha_1^2 = 0.1, \alpha_2 = 0, \beta^2 = 0.3, \gamma^2 = 0$. In this and the following two figures, red dots mark the proportion of simulated data where the 95% confidence interval for γ contained the true γ value. The error bars represent the mean±SE of the proportion estimate. SE was calculated as $\sqrt{q \cdot (1-q)/d}$, where q is the mean proportion (when the value falls into the 95% confidence interval) and d is the number of generated data sets (500 in these examples).

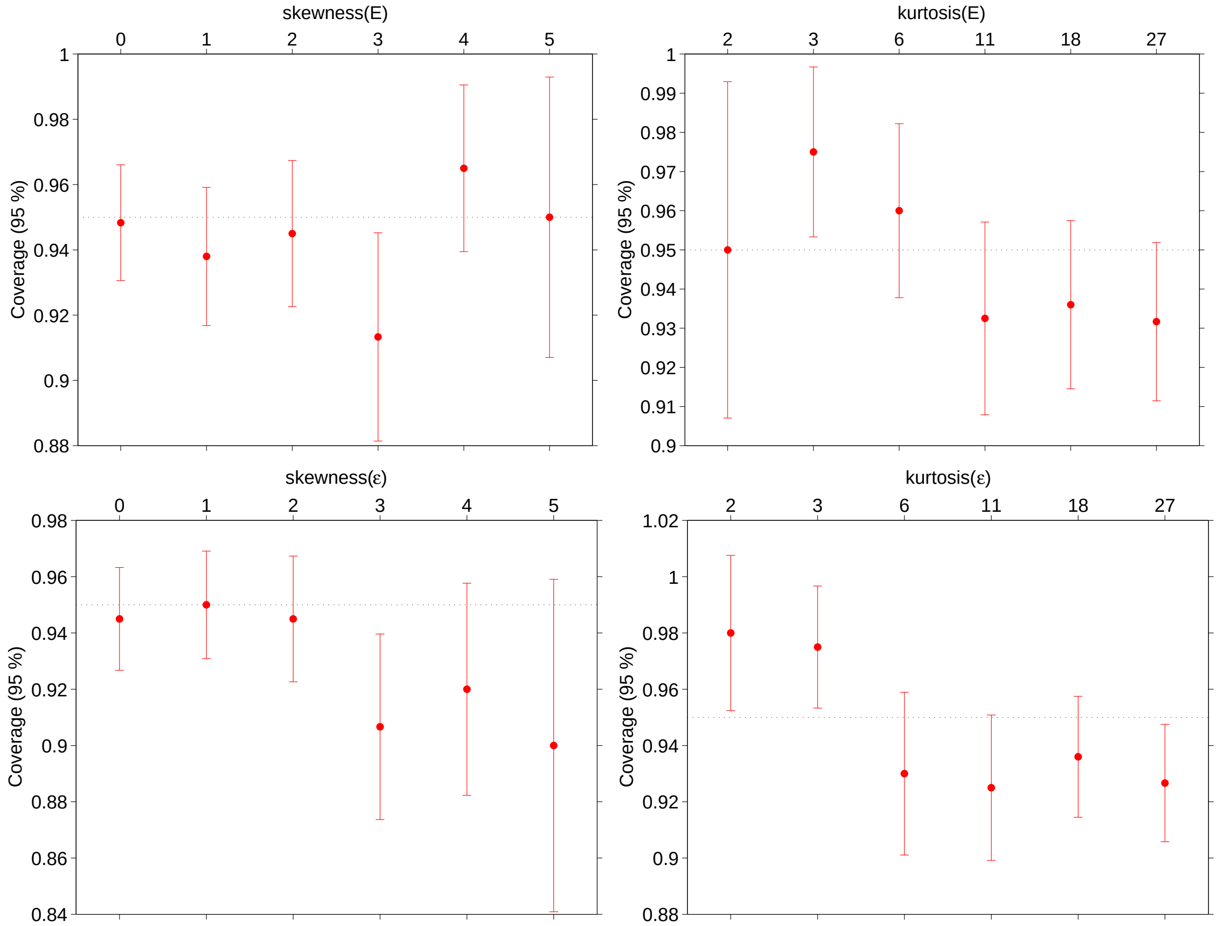

Supplementary Figure 5: Coverage of the 95% confidence interval as a function of skewness and kurtosis of the environmental variable (E - top panels) or the noise (ε - bottom panels). Parameters were fixed as above: $n = 1,000, \alpha_1^2 = 0.1, \alpha_2 = 0, \beta^2 = 0.3, \gamma^2 = 0$. The error bars represent the mean±SE of the proportion estimate from 500 simulated data sets

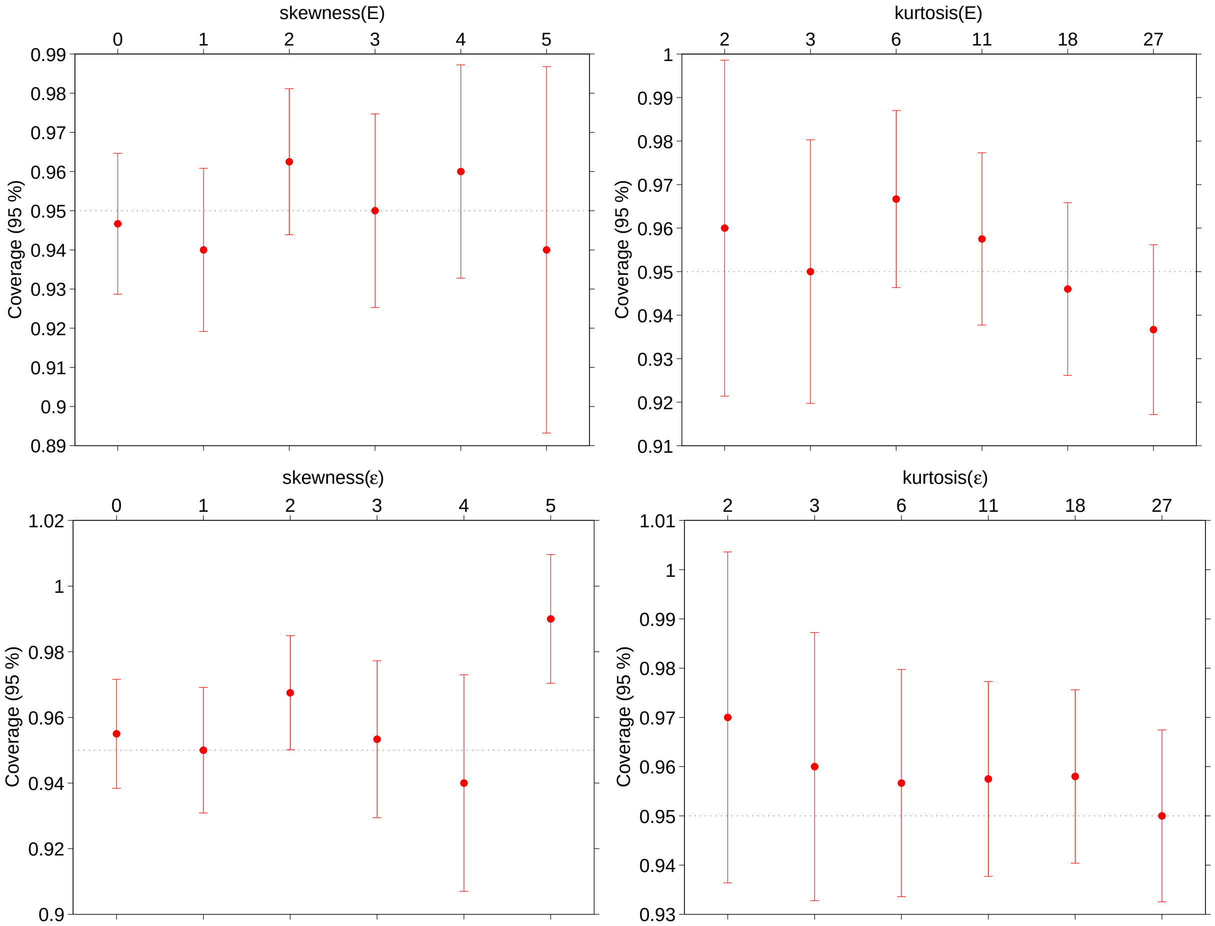

Supplementary Figure 6: Coverage of the 95% confidence interval as a function of skewness and kurtosis of the environmental variable (E - top panels) or the noise (ε - bottom panels). Parameters were fixed as above: $n = 10,000, \alpha_1^2 = 0.1, \alpha_2 = 0, \beta^2 = 0.3, \gamma^2 = 0.025$. The error bars represent the mean±SE of the proportion estimate from 500 simulated data sets.

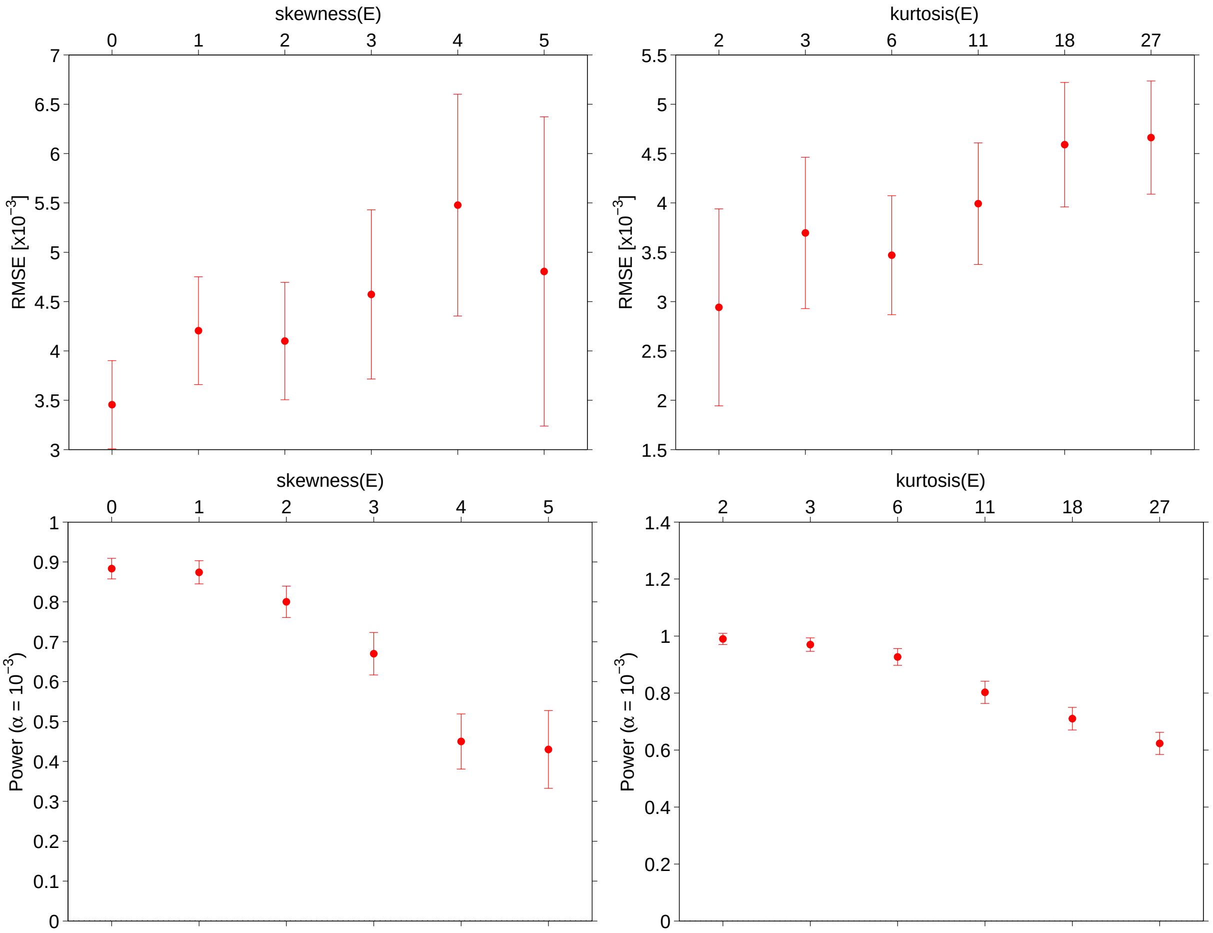

Supplementary Figure 7: Root mean square error (RMSE) and power (at 10^{-3}) of the interaction effect estimation as a function of skewness and kurtosis of the environmental variable (E). Red dots represent the mean±SE of the RMSE/power over the 500 generated data sets.

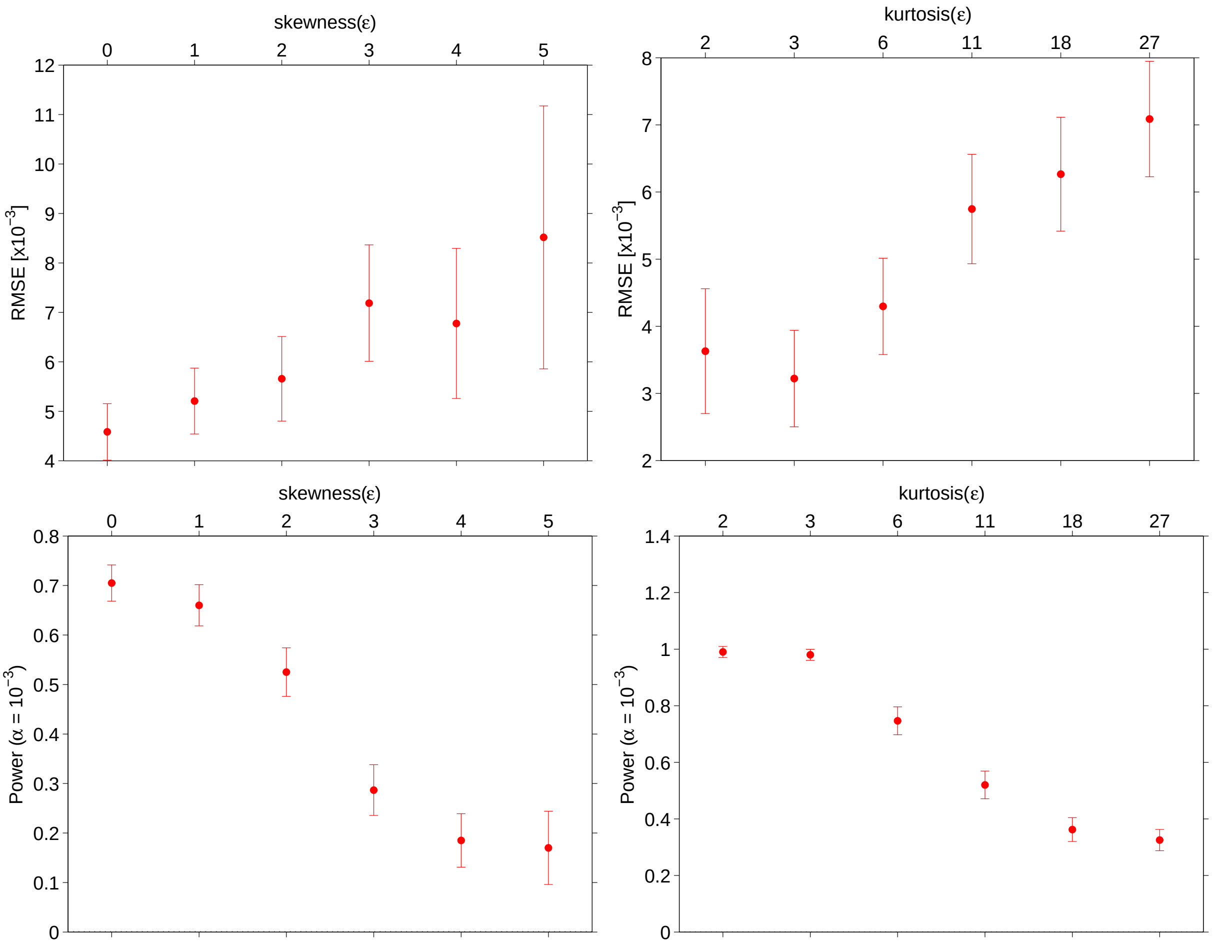

Supplementary Figure 8: Root mean square error (RMSE) and power (at 10^{-3}) of the interaction effect estimation as a function of skewness and kurtosis of the residual noise term (ε). Red dots represent the mean±SE of the RMSE/power over the 500 generated data sets.

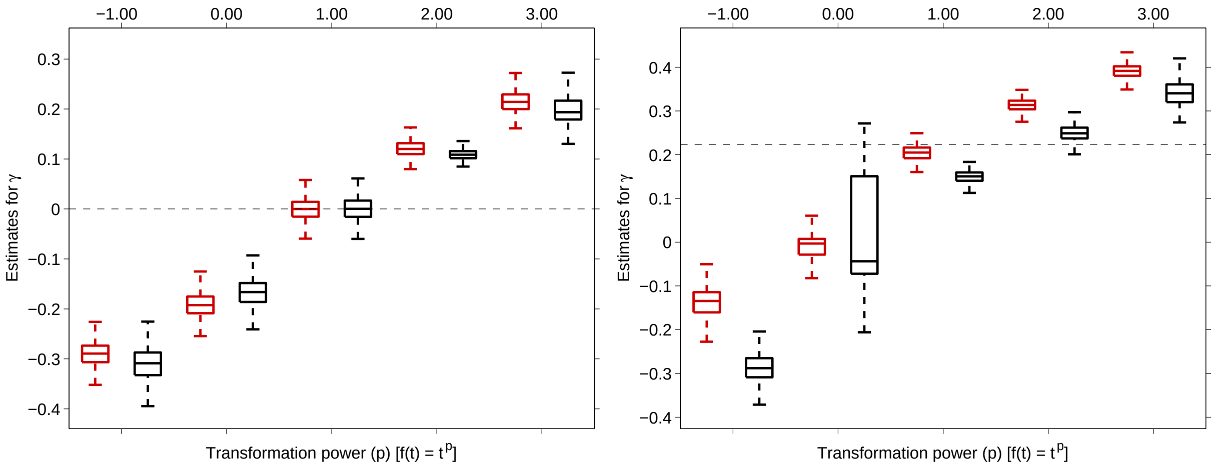

Supplementary Figure 9: The impact of more extreme transformations on the estimation for **GRS** and **fGRS**. Left panel for $\gamma = 0$, right panel for $\gamma = \sqrt{0.05} \approx 0.22$. Boxplots are based on 500 simulated data sets and boxes mark the first (q_1) second (q_2) and third quartiles (q_3) and the lower/upper whiskers are at $q_1 - 1.5 \cdot (q_3 - q_1)$, $q_3 + 1.5 \cdot (q_3 - q_1)$, respectively.

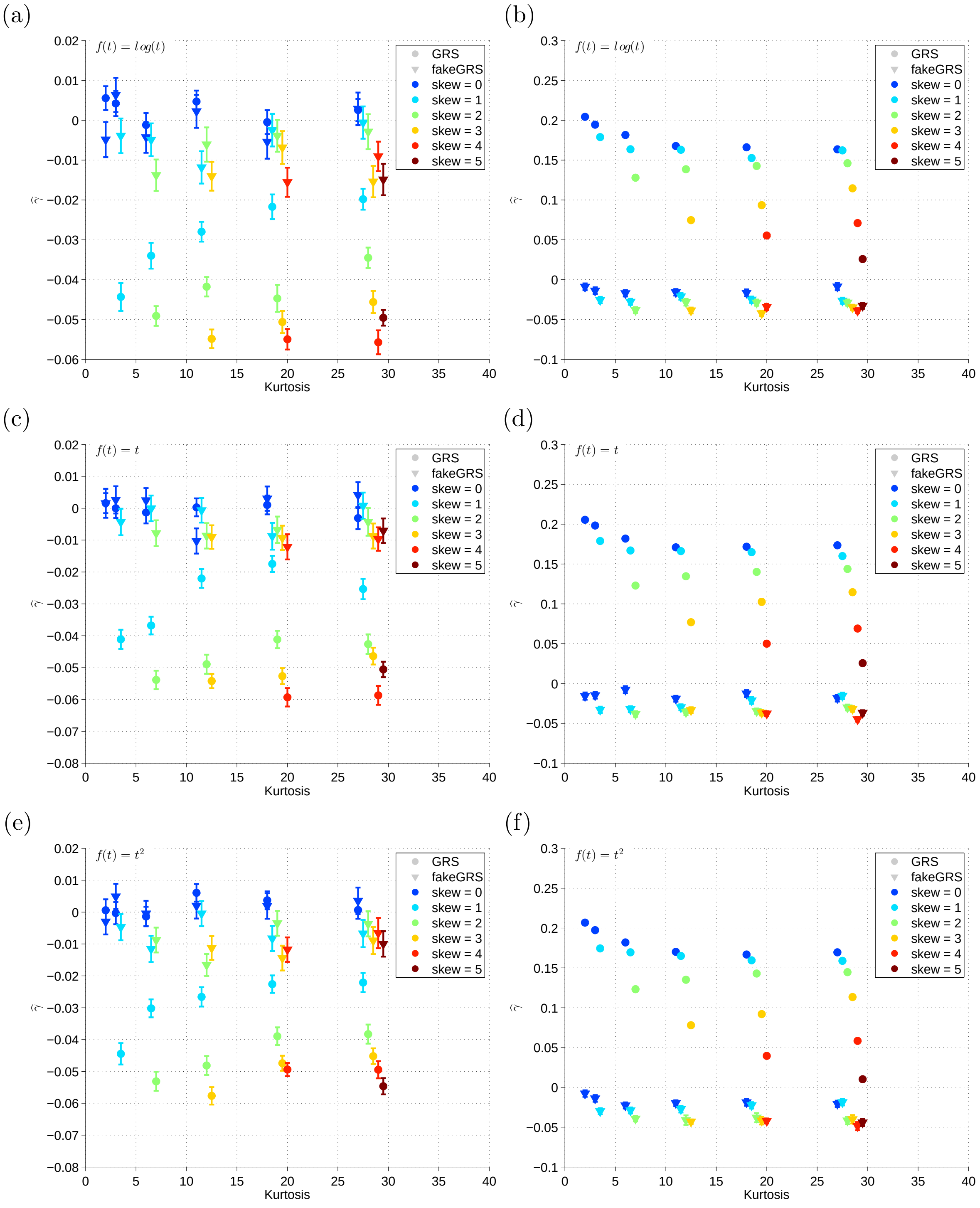

Supplementary Figure 10: Estimations for inverse normal quantile transformed outcomes. Error bars (mean± SE) for the interaction effect estimates ($\widehat{\gamma}$) for the real data ($\bullet$) and for fake G ($\bigtriangledown$) as a function of transformation power ($p \in \{0, 1, 2\} : f(t) = t^p$) (rows 1-3). Other parameters were fixed at $n = 10,000$, $\alpha_1^2 = 0.05$, $\alpha_2 = 0$, and $\beta^2 = 0.3$. **Left column** without GxE interaction ($\gamma = 0$), **Right column**: With GxE interaction ($\gamma = \sqrt{0.05} \approx 0.22$).

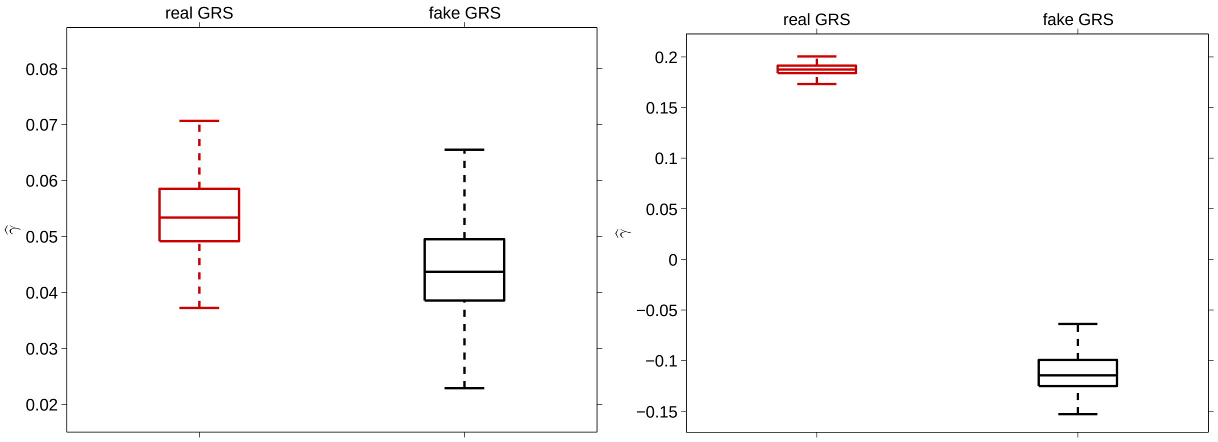

Supplementary Figure 11: Estimates for data simulation settings imitating UK Biobank parameters for BMI (left panel) and leg impedance (right panel). We set parameters for BMI as $\alpha_1 = 0.23, \beta = 0.65, \gamma = 0, f(t) = t^2$ and for leg impedance $\alpha_1 = 0.20, \beta = 0.45, \gamma = 0.2, f(t) = log(t)$. Boxplots are based on 500 simulated data sets and boxes mark the first (q_1) second (q_2) and third quartiles (q_3) and the lower/upper whiskers are at $q_1 - 1.5 \cdot (q_3 - q_1)$, $q_3 + 1.5 \cdot (q_3 - q_1)$, respectively.

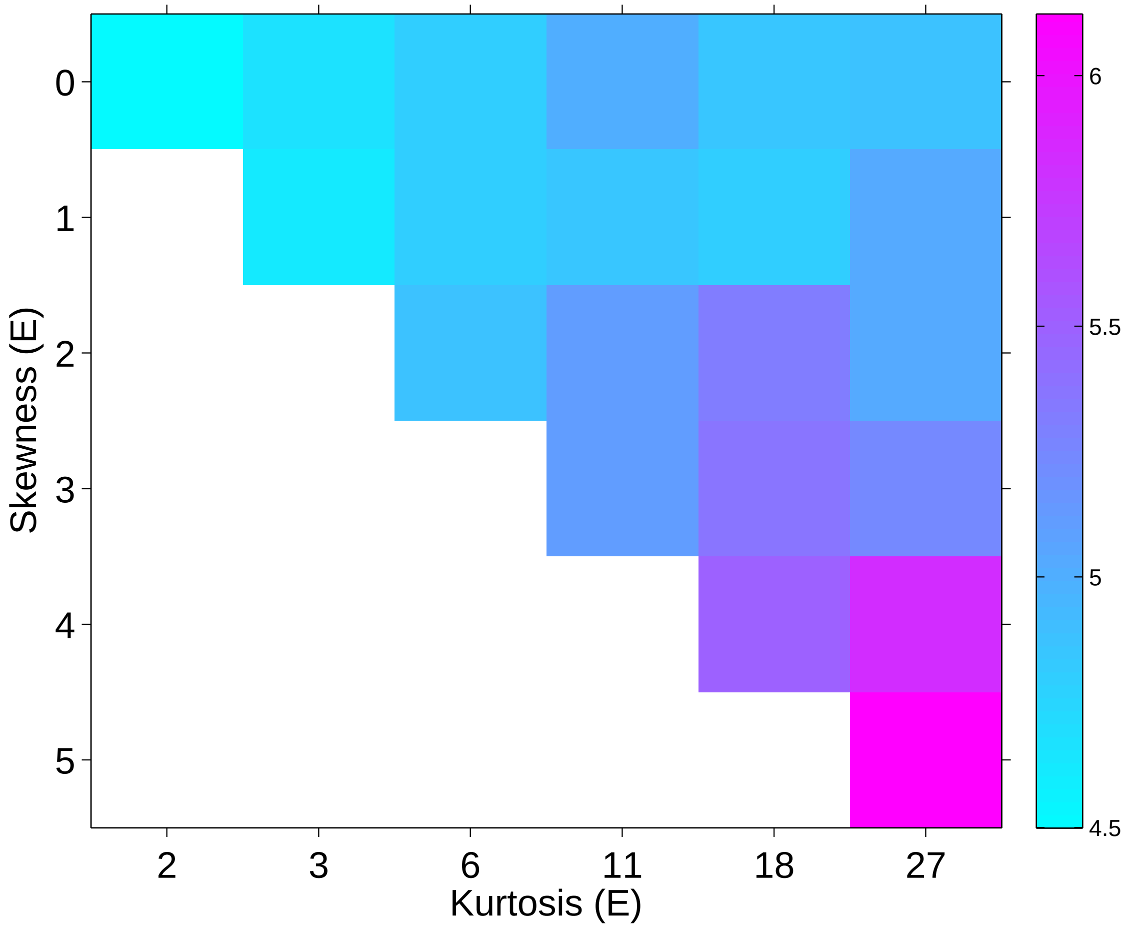

Supplementary Figure 12: Ratio of the test statistics from linear regression with interaction term when E is observed and the test statistic from our ML-based estimation with unobserved E for various skewness and kurtosis values for E. We used the following settings: $n = 10,000, \alpha_1 = \sqrt{0.05}, \beta = \sqrt{0.3}, \gamma = \sqrt{0.05}$.

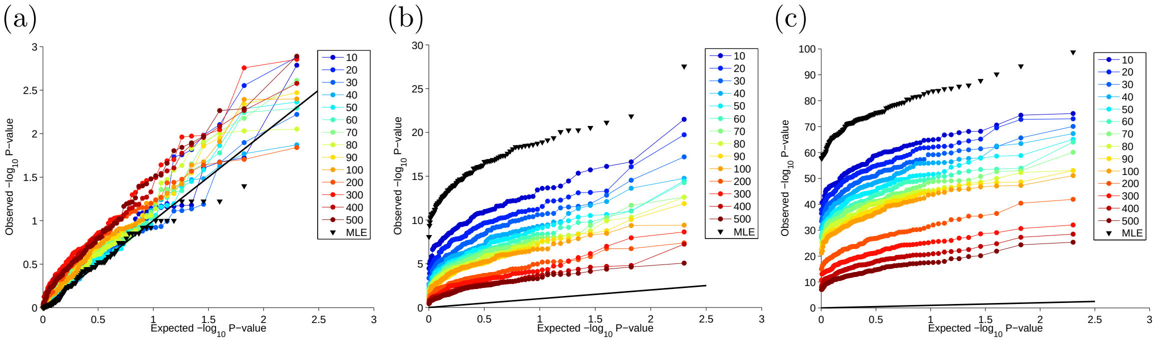

Supplementary Figure 13: QQ-plots of the P-values obtained over 100 data generations using the Brown-Forsythe test in comparison with our method. Since the Brown-Forsythe test needs grouped data we applied various numbers of bins ranging from 10 to 500, indicated by the different colours. (Bins lower than ten lead to similar results as 10 bins.) We used the following settings: $n = 10,000, \alpha_1 = \sqrt{0.05}, \beta = \sqrt{0.3}$, $\gamma = 0, \sqrt{0.01}, \sqrt{0.05}$ for panels (a), (b) and (c), respectively. The (median) power increase in case of $\gamma = \sqrt{0.01}$ / $\gamma = \sqrt{0.05}$ is equivalent to $\approx$ 50% / 33% increase in sample size.

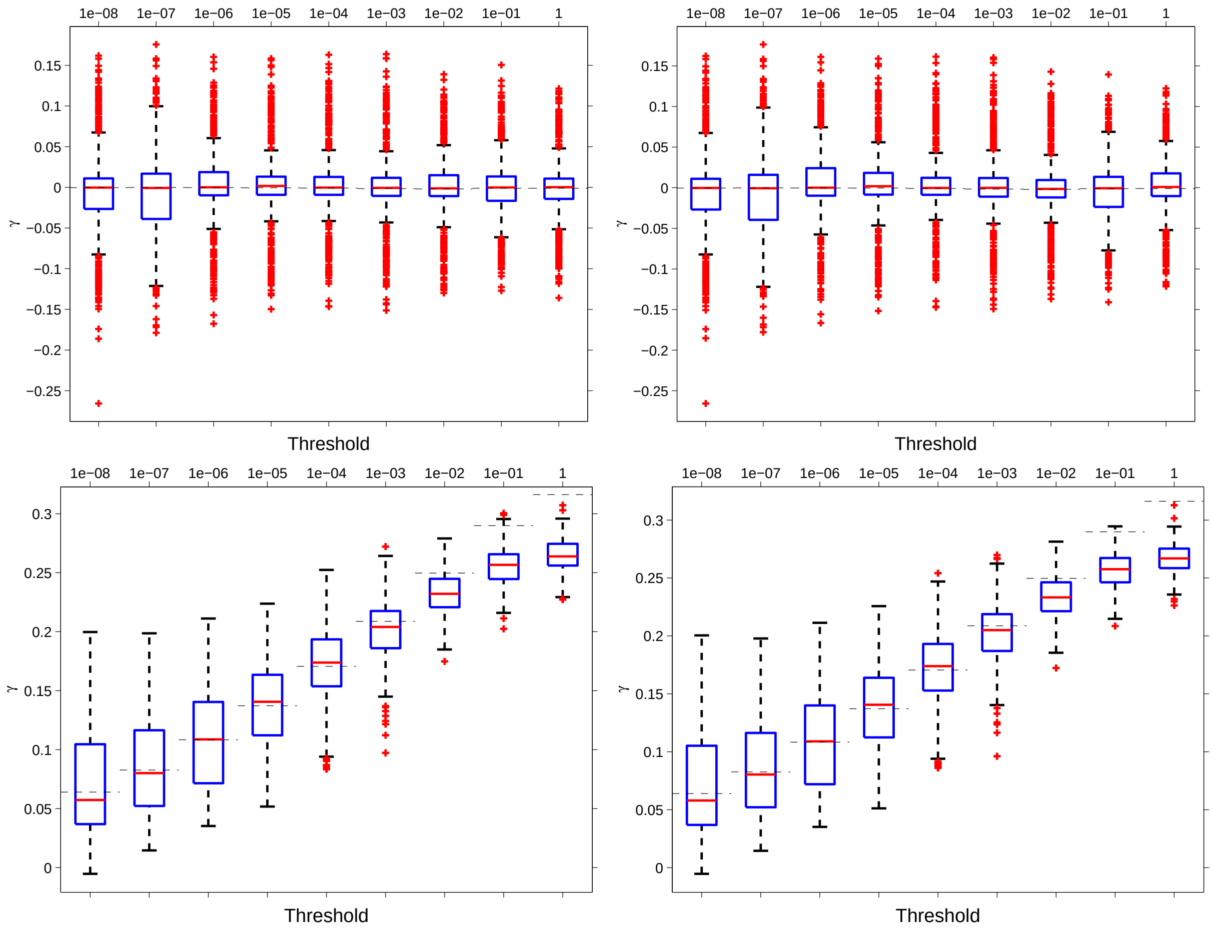

Supplementary Figure 14: Box plots of the 500 interaction parameter estimates ($\widehat{\gamma}$) for estimated GRSs. Models are defined as before and the true GRS is $\boldsymbol{g} = \boldsymbol{G} \cdot \pi$ with $\pi \sim \mathcal{N}(0, h^2/m)$, where m is the number of causal markers. In our challenge $\boldsymbol{g}$ was then masked, only $\boldsymbol{G}$ is observed in addition to $\boldsymbol{y}$. GRS was estimated via least squares regression (left column) or via BLUP (right column). We derived GRSs at different P-value thresholds ranging from 10^{-8} to 1. The horizontal dashed lines in the bottom panel represent the median estimate for the GRS when selecting only SNPs with P-value below the indicated threshold, but their true coefficients is assumed to be known. The boxplots represent the interaction effect estimates (based on 100 data generations) for the GRS for the same set of SNPs, but their coefficients are estimated from the data. Parameters are set to $n = 10,000, m = 1,000, \alpha^2 = 0.3, \beta^2 = 0.3$, $\gamma^2 = 0$ (top row) / $\gamma^2 = 0.1$ (bottom row). One can observe that using the estimated GRSs (instead of the true values) does not noticeably bias the interaction estimates for reasonable ($< 10^{-3}$) P-value thresholds, but the estimates become conservative for more liberal SNP selection. The underlying γ value can only be reached if all SNPs are used (last bin). BLUP estimator was defined as $\widehat{\beta}_{\text{BLUP}} = \sigma_g^2 \boldsymbol{G}'(\sigma_g^2 \boldsymbol{G}\boldsymbol{G}'/m + \sigma_\varepsilon^2 I)^{-1}\boldsymbol{y}$. Boxes mark the first ($q_1$) second ($q_2$) and third quartiles ($q_3$) and the lower/upper whiskers are at $q_1 - 1.5 \cdot (q_3 - q_1)$, $q_3 + 1.5 \cdot (q_3 - q_1)$, respectively.

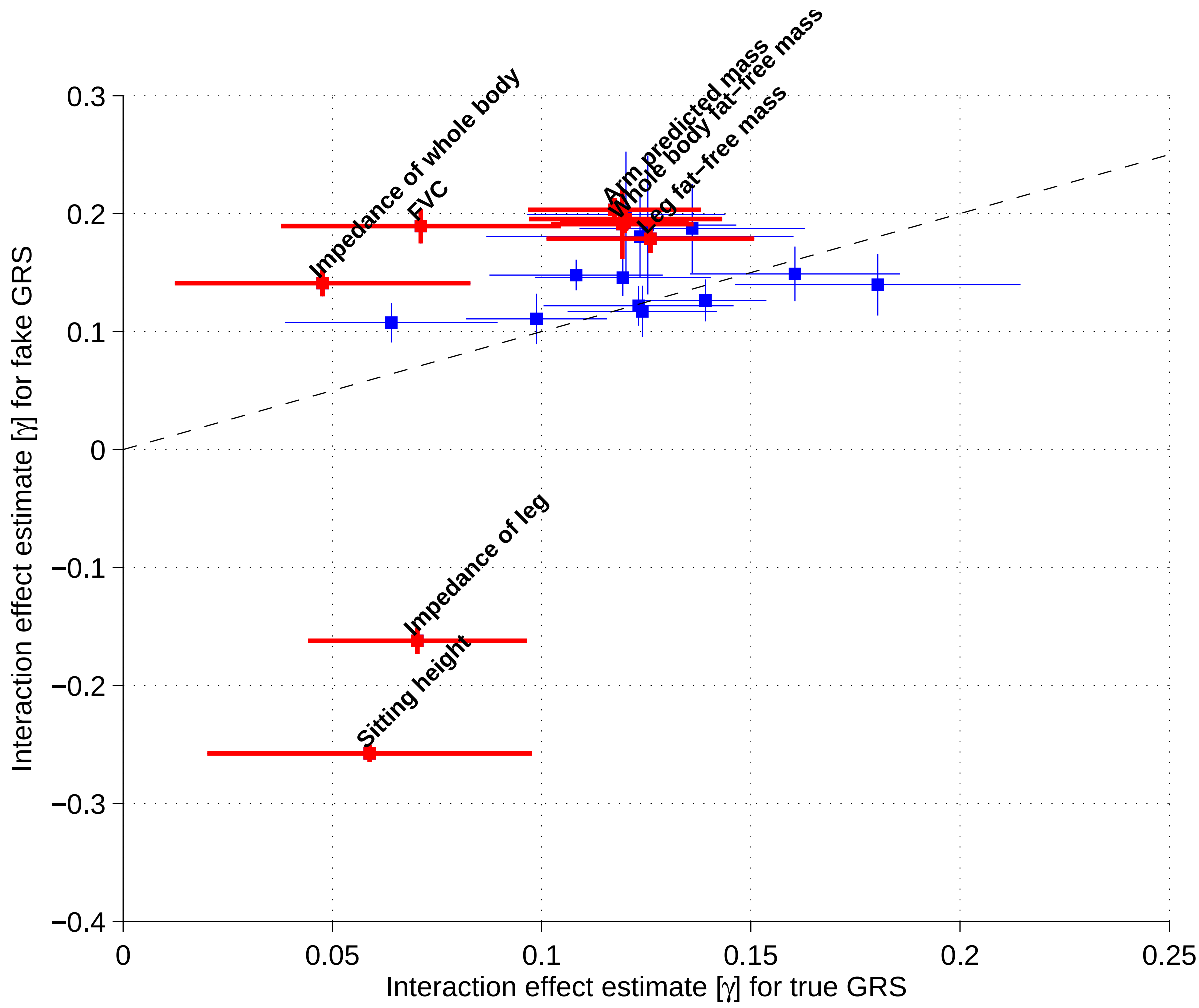

Supplementary Figure 15: Estimated GRSxE interaction effects are shown for 22 continuous traits measured in the UK Biobank ($n = 378,836$). The x-axis position of each dot (trait) represents the GxE estimate for the GRS, while the y-axis location corresponds to the interaction effect for the counterfeit GRS. Error bars indicate the mean and the 95% confidence intervals, those in red remain significant after Bonferroni correction at 5% type I error level when testing the difference between the two estimates. Labels for trunk predicted mass, trunk fat-free mass were omitted as they overlap with arm predicted mass and whole body fat-free mass, respectively.

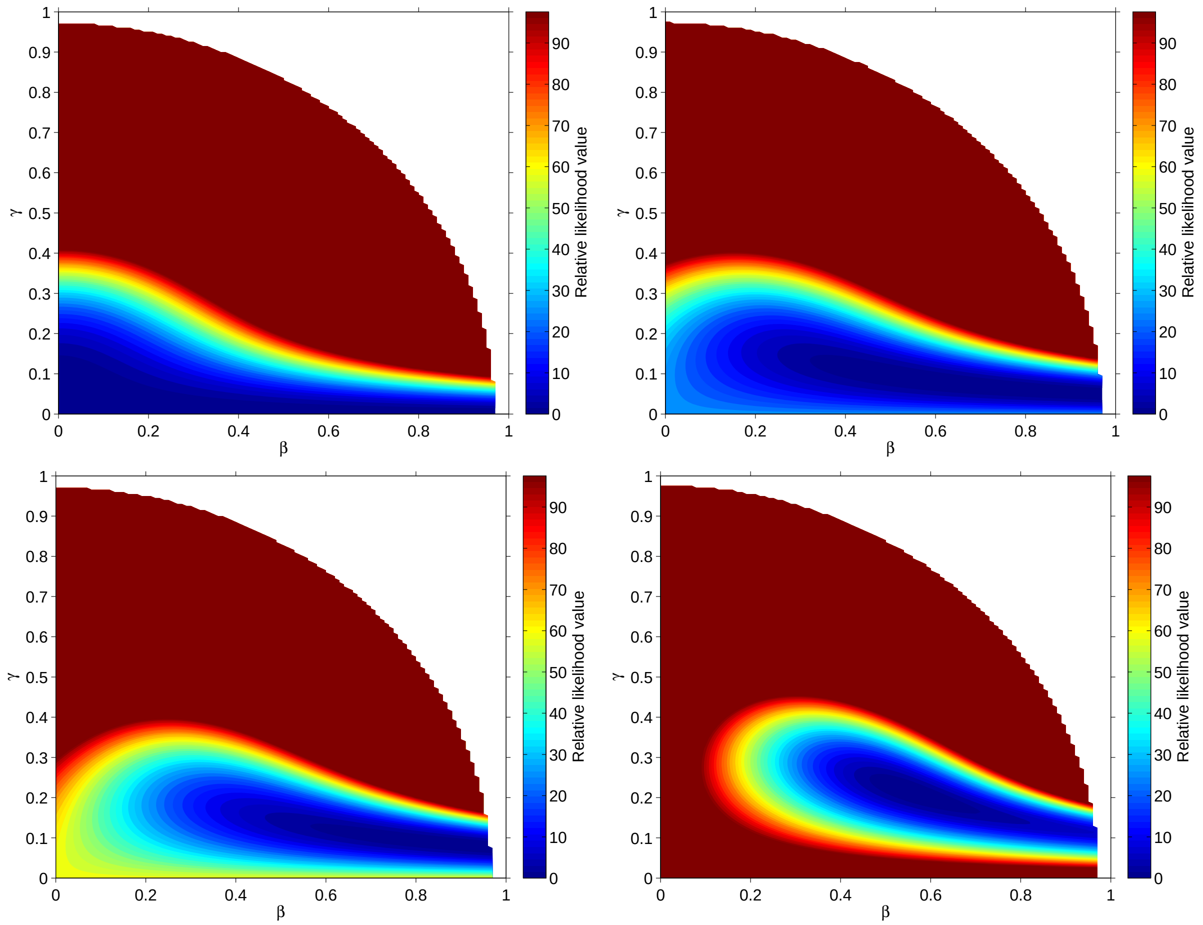

Supplementary Figure 16: The negative log-likelihood surface contour plot for various values of interaction effects γ. In case of small interaction effect, the marginal environmental effect size β is not identifiable and the optimum parameters end up on the constraint boundary. As interaction effect size (or sample size) increases, the fraction of datasets leading to an optimum sitting on the boundary decreases. For this analysis we used untransformed trait, $n = 10,000$, $\alpha = \sqrt{0.05}, \beta = \sqrt{0.3}$, $\gamma = 0$ (top left), $\sqrt{0.01}$ (top right), $\sqrt{0.025}$ (bottom left), $\sqrt{0.05}$ (bottom right). These settings led to 50%, 27%, 4% and 0% optima ending up on the boundary, respectively.

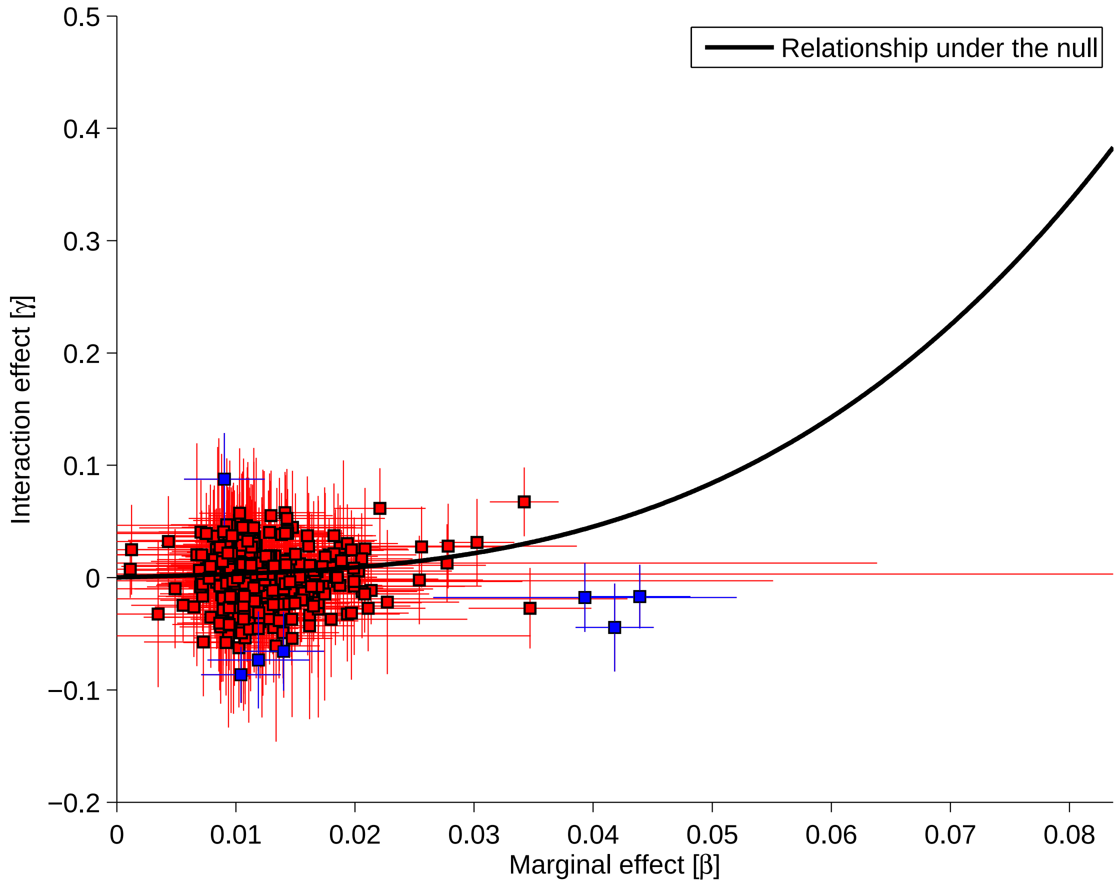

Supplementary Figure 17: Interaction vs marginal effects for 361 leg impedance-associated SNPs. Black line represents the relationship for a non-specific variable. Only seven SNPs show significantly different effect than expected.

Supplementary Tables

Trait	Skewness	Kurtosis
Sitting height	0.0145	3.7420
Body mass index (BMI)	1.0918	5.4744
Weight	0.7629	4.2034
Whole body fat mass	1.1370	5.4759
Whole body fat-free mass	0.5246	2.4007
Whole body water mass	0.5329	2.4386
Basal metabolic rate	0.6752	2.9897
Impedance of whole body	0.2279	2.8387
Impedance of leg	0.2622	4.5960
Leg fat percentage	-0.0713	1.8521
Leg fat mass	1.2804	6.8168
Leg fat-free mass	0.6672	3.0732
Leg predicted mass	0.6538	3.0121
Arm fat percentage	0.5704	2.6979
Arm fat mass	2.3575	15.4061
Arm fat-free mass	0.6780	3.8729
Arm predicted mass	0.6703	3.8760
Trunk fat-free mass	0.4789	2.2962
Trunk predicted mass	0.4729	2.2796
FVC	1.6167	30.6670
Waist circumference	0.4297	3.3335
Hip circumference	1.1080	6.1206

Supplementary Table 1: Skewness and kurtosis values are shown for the 22 (of the 32) continuous traits measured in the UK Biobank for which no maximum likelihood estimation convergence issues were detected.

**Trait**	$\widehat{\alpha_1}$	$\widehat{\alpha_2}$	$\widehat{\beta}$	$\widehat{\gamma}(P)$	$\widehat{\gamma_K}(P)$	P_Δ
Leg fat mass	0.215	0.005	0.98	0.022 (2.3e-100)	0.003 (8.9e-01)	4.5e-01
FVC	0.226	0.004	0.97	0.024 (5.2e-95)	0.018 (3.5e-01)	7.3e-01
FEV1	0.191	0.002	0.98	0.025 (5.2e-87)	0.031 (5.4e-02)	7.0e-01
Whole body fat mass	0.226	0.004	0.97	0.019 (2.8e-59)	-0.000 (9.9e-01)	3.6e-01
Trunk fat mass	0.226	0.003	0.97	0.017 (1.0e-52)	0.024 (1.4e-01)	6.8e-01
Hip circumference	0.224	0.003	0.97	0.016 (1.0e-49)	0.021 (2.3e-01)	7.8e-01
Arm fat mass	0.219	0.004	0.98	0.018 (1.3e-49)	0.030 (3.9e-02)	4.3e-01
Birth weight	0.155	0.002	0.98	0.021 (1.8e-28)	0.023 (2.5e-01)	9.1e-01
Trunk fat percentage	0.204	0.004	0.98	0.010 (1.3e-18)	0.025 (1.3e-01)	3.5e-01
Sitting height	0.363	0.001	0.93	0.010 (1.5e-18)	0.004 (8.6e-01)	8.0e-01
Standing height	0.450	0.004	0.89	0.009 (2.1e-16)	0.000 (1.0e+00)	6.9e-01
Arm predicted mass	0.274	0.000	0.96	0.008 (7.1e-11)	-0.002 (9.4e-01)	7.0e-01
Arm fat-free mass	0.275	0.000	0.96	0.007 (2.2e-09)	-0.001 (9.8e-01)	7.3e-01
Trunk fat-free mass	0.312	0.001	0.95	0.005 (1.8e-06)	-0.003 (9.1e-01)	7.4e-01
Impedance of leg	0.256	-0.001	0.96	0.007 (1.2e-04)	-0.000 (9.9e-01)	7.3e-01
Waist circumference	0.197	0.006	0.87	0.023 (9.3e-03)	0.036 (1.9e-02)	4.7e-01
Whole body water mass	0.306	0.000	0.95	0.003 (1.8e-02)	-0.007 (7.8e-01)	6.8e-01
Body mass index (BMI)	0.233	0.004	0.85	0.021 (2.0e-02)	0.024 (1.1e-01)	8.4e-01
Pulse rate	0.220	-0.002	0.91	-0.015 (2.2e-02)	0.020 (2.8e-01)	7.4e-02
Leg fat percentage	0.203	0.003	0.87	0.020 (2.5e-02)	0.022 (1.9e-01)	9.4e-01
Weight	0.261	0.002	0.90	0.012 (4.2e-02)	0.019 (2.5e-01)	7.1e-01
Arm fat percentage	0.205	0.004	0.47	0.037 (4.2e-02)	0.020 (2.2e-01)	4.9e-01
Body fat percentage	0.210	0.004	0.79	0.021 (5.4e-02)	0.019 (3.3e-01)	9.3e-01
Whole body fat-free mass	0.306	-0.000	0.95	0.002 (5.4e-02)	0.016 (3.6e-01)	4.3e-01
Impedance of arm	0.247	0.001	0.95	-0.002 (7.7e-02)	-0.001 (9.6e-01)	9.7e-01
Trunk predicted mass	0.312	0.001	0.93	0.006 (8.7e-02)	0.023 (1.5e-01)	2.9e-01
Basal metabolic rate	0.293	0.001	0.94	0.004 (2.1e-01)	0.004 (8.6e-01)	9.8e-01
Impedance of whole body	0.265	0.002	0.91	0.001 (3.3e-01)	0.020 (3.0e-01)	3.3e-01
Leg fat-free mass	0.283	-0.000	0.90	0.001 (4.5e-01)	0.027 (3.4e-02)	4.1e-02
Leg predicted mass	0.282	0.000	0.91	0.001 (5.0e-01)	0.025 (1.3e-01)	1.4e-01
Systolic blood pressure	0.169	-0.003	0.91	0.002 (5.5e-01)	-0.026 (9.3e-02)	7.7e-02
Diastolic blood pressure	0.167	-0.004	0.49	-0.012 (5.5e-01)	-0.026 (1.0e-01)	6.0e-01
WHR	0.201	-0.002	0.26	0.001 (7.1e-01)	0.401 (8.8e-11)	9.8e-11

Supplementary Table 2: Estimated contribution of GRSxE effects are shown all 32 **inverse normal quantile transformed** continuous traits measured in the UK Biobank . Column label abbreviations are as follows: α_1: GRS effect, α_2: GRS^2 effect, β : environmental effect, γ : interaction effect, γ_K : interaction effect of fake GRS, P_Δ : P-value for testing $\gamma = \gamma_K$. Note that significantly non-zero interactions are claimed only when the P-value of the estimate ($\widehat{\gamma}$) is below 0.05/32, i.e. $P_\gamma < 1.5 \times 10^{-3}$, which is based on the Bonferroni correction for multiple testing (ensuring family wise error rate control at 5%). P_γ was calculated based on the Wald test (2-sided) and P_Δ was derived from a two-sided Z-test.

**Trait**	α	β	γ	α_F	β_F	γ_F	**skew**	**kurtosis**	**FitRes**
Body mass index (BMI)	0.23	0.64	0.14	0.23	0.65	0.14	-0.20	3.04	0.00
Impedance of leg (right)	0.25	0.08	0.07	0.25	0.13	0.12	-0.20	3.04	0.01
Trunk predicted mass	0.31	0.31	0.12	0.31	0.24	0.14	0.00	3.00	0.01
Whole body fat-free mass	0.30	0.38	0.12	0.30	0.34	0.14	0.00	3.00	0.01
Whole body fat mass	0.22	0.70	0.13	0.22	0.72	0.13	-0.60	4.36	0.01
Leg fat mass (right)	0.21	0.67	0.16	0.21	0.69	0.15	-0.20	3.04	0.01
Whole body water mass	0.30	0.37	0.13	0.30	0.36	0.14	0.00	3.00	0.00
Trunk fat-free mass	0.31	0.31	0.12	0.31	0.25	0.14	0.00	3.00	0.00
Arm predicted mass (right)	0.27	0.39	0.12	0.27	0.35	0.14	0.00	3.00	0.01
Weight	0.25	0.65	0.13	0.25	0.65	0.14	-0.20	3.04	0.00
Arm fat mass (right)	0.21	0.70	0.17	0.20	0.72	0.19	-0.40	3.16	0.02
Arm fat percentage (right)	0.20	0.25	0.11	0.20	0.28	0.11	-0.20	3.04	0.01
Leg fat-free mass (right)	0.27	0.53	0.13	0.27	0.62	0.14	-0.40	4.16	0.01
Arm fat-free mass (right)	0.27	0.39	0.12	0.27	0.35	0.14	0.00	3.00	0.01
Basal metabolic rate	0.29	0.46	0.14	0.29	0.48	0.14	0.00	3.00	0.01
Leg predicted mass (right)	0.27	0.52	0.13	0.27	0.63	0.14	-0.40	4.16	0.01
Leg fat percentage (right)	0.20	0.43	0.06	0.20	0.33	0.08	-0.20	3.04	0.01
Forced vital capacity (FVC)	0.22	0.54	0.06	0.22	0.29	0.06	-0.20	4.04	0.01
Sitting height	0.36	0.07	0.06	0.35	0.07	0.09	-0.20	4.04	0.02
Impedance of whole body	0.26	0.20	0.05	0.26	0.25	0.11	-0.20	3.04	0.01
Waist circumference	0.20	0.63	0.10	0.20	0.62	0.10	-0.20	3.04	0.01
Hip circumference	0.22	0.68	0.12	0.22	0.69	0.11	-0.40	4.16	0.02

Supplementary Table 3: Estimated contribution of GRSxE effects are shown for the 22 selected continuous traits measured in the UK Biobank and compared to the estimates obtained for a counterfeit transformed outcome. Column label abbreviations are as follows: α: GRS effect, β : environmental effect, γ : interaction effect, α_F : GRS effect on fake Y, β_F : environmental effect on fake Y,γ_F : interaction effect on fake Y, skew: skewness of the error term ϵ, kurtosis: kurtosis of ϵ, FitRes: root mean square difference between the fake phenotype $\boldsymbol{y}^\star := f(\boldsymbol{z})$ and the real phenotype $(\boldsymbol{y})$.
