# Supplementary material for: Quantification of the overall contribution of gene-environment interaction for obesity-related traits
Source: Nat Commun. 2020 Mar 13;11:1385. doi: 10.1038/s41467-020-15107-0 (PMC7070002; doi:10.1038/s41467-020-15107-0)
Supplement: Supplementary file 3 — Reporting Summary [file 41467_2020_15107_MOESM3_ESM.pdf]

nature research

Corresponding author(s): Zoltan Kutalik
NCOMMS-19-21103

Last updated by author(s): Jan 31, 2020

Reporting Summary

Nature Research wishes to improve the reproducibility of the work that we publish. This form provides structure for consistency and transparency in reporting. For further information on Nature Research policies, see Authors & Referees and the Editorial Policy Checklist.

Statistics

For all statistical analyses, confirm that the following items are present in the figure legend, table legend, main text, or Methods section.

n/a	Confirmed	
☐	☒	The exact sample size (*n*) for each experimental group/condition, given as a discrete number and unit of measurement
☐	☒	A statement on whether measurements were taken from distinct samples or whether the same sample was measured repeatedly
☐	☒	The statistical test(s) used AND whether they are one- or two-sided<br>*Only common tests should be described solely by name; describe more complex techniques in the Methods section.*
☐	☒	A description of all covariates tested
☐	☒	A description of any assumptions or corrections, such as tests of normality and adjustment for multiple comparisons
☐	☒	A full description of the statistical parameters including central tendency (e.g. means) or other basic estimates (e.g. regression coefficient) AND variation (e.g. standard deviation) or associated estimates of uncertainty (e.g. confidence intervals)
☐	☒	For null hypothesis testing, the test statistic (e.g. *F*, *t*, *r*) with confidence intervals, effect sizes, degrees of freedom and *P* value noted<br>*Give P values as exact values whenever suitable.*
☐	☒	For Bayesian analysis, information on the choice of priors and Markov chain Monte Carlo settings
☐	☒	For hierarchical and complex designs, identification of the appropriate level for tests and full reporting of outcomes
☐	☒	Estimates of effect sizes (e.g. Cohen's *d*, Pearson's *r*), indicating how they were calculated

Our web collection on statistics for biologists contains articles on many of the points above.

Software and code

Policy information about availability of computer code

Data collection	All data were either simulated (code provided) or provided by the UK Biobank resource (#16389)
Data analysis	The code was written in the R /Matlab programming languages. Respective codes are available on the following GitHub page https://github.com/zkutalik/GRSxE_software

For manuscripts utilizing custom algorithms or software that are central to the research but not yet described in published literature, software must be made available to editors/reviewers. We strongly encourage code deposition in a community repository (e.g. GitHub). See the Nature Research guidelines for submitting code & software for further information.

Data

Policy information about availability of data

All manuscripts must include a data availability statement. This statement should provide the following information, where applicable:

- Accession codes, unique identifiers, or web links for publicly available datasets
- A list of figures that have associated raw data
- A description of any restrictions on data availability

UK Biobank data are available upon approved application. Simulated data can be produced by executing the provided code.

Field-specific reporting

Please select the one below that is the best fit for your research. If you are not sure, read the appropriate sections before making your selection.

☒ Life sciences ☐ Behavioural & social sciences ☐ Ecological, evolutionary & environmental sciences

Life sciences study design

All studies must disclose on these points even when the disclosure is negative.

Sample size	To maximise power, we used all samples provided by the UK Biobank.
Data exclusions	Non-British individuals (to reduce false positive associations due to population stratification), or those with missing data were excluded from the analysis.
Replication	No sufficiently large other data set was available for replication.
Randomization	We had no experimental groups.
Blinding	There was no group allocation.

Reporting for specific materials, systems and methods

We require information from authors about some types of materials, experimental systems and methods used in many studies. Here, indicate whether each material, system or method listed is relevant to your study. If you are not sure if a list item applies to your research, read the appropriate section before selecting a response.

Materials & experimental systems

n/a	Involved in the study	
☒	☐	Antibodies
☒	☐	Eukaryotic cell lines
☒	☐	Palaeontology
☒	☐	Animals and other organisms
☐	☒	Human research participants
☒	☐	Clinical data

Methods

n/a	Involved in the study	
☒	☐	ChIP-seq
☒	☐	Flow cytometry
☒	☐	MRI-based neuroimaging

Human research participants

Policy information about studies involving human research participants

Population characteristics	Described in the UK Biobank protocol: https://www.ukbiobank.ac.uk/wp-content/uploads/2011/11/UK-Biobank-Protocol.pdf In brief, UK Biobank consists of ~500,000 men and women from the UK general population aged 40 to 69. The UKB sample
Recruitment	BMI is slightly lower (27. 4+/-4.8 SD) than the general population, which may introduce mild selection bias, which might affect the results.
Ethics oversight	The UK biobank study was approved by the National Research Ethics Service Committee.

Note that full information on the approval of the study protocol must also be provided in the manuscript.
